# WS-5 Extract of* Curcuma longa, Chaenomeles sinensis,* and* Zingiber officinale* Contains Anti-AChE Compounds and Improves *β*-Amyloid-Induced Memory Impairment in Mice

**DOI:** 10.1155/2019/5160293

**Published:** 2019-04-01

**Authors:** Ju Eun Kim, Abinash Chandra Shrestha, Hyo Shin Kim, Ha Neul Ham, Jun Hyeong Kim, Yeong Jee Kim, Yun Jeong Noh, Su Jin Kim, Dae Keun Kim, Hyung Kwon Jo, Dae Sung Kim, Kwang Hyun Moon, Jeong Ho Lee, Kyung Ok Jeong, Jae Yoon Leem

**Affiliations:** ^1^College of Pharmacy, Woosuk University, Wanju, Jeonbuk 55338, Republic of Korea; ^2^Hanpoong Pharm. Co., LTD, Wanju, Jeonbuk, 55336, Republic of Korea; ^3^Sunchang Institute of Health and Longevity, Sunchang, Jeonbuk 56015, Republic of Korea

## Abstract

Alzheimer's disease (AD) is linked to an extensive neuron loss via accumulation of amyloid-beta (A*β*) as senile plaques associated with reactive astrocytes and microglial activation in the brain. The objective of this study was to assess the therapeutic effect of WS-5 ethanol extract in vitro and in vivo against A*β*-induced AD in mice and to identify the extract's active constituents. In the present study, WS-5 exerted a significant inhibitory effect on acetylcholinesterase (AChE). Analysis by transmission electron microscopy (TEM) revealed that WS-5 prevented A*β* oligomerization via inhibition of A*β*_1-42_ aggregation. Evaluation of antioxidant activities using 1, 1-diphenyl-2-picrylhydrazyl (DPPH) demonstrated that WS-5 possessed a high antioxidant activity, which was confirmed by measuring the total antioxidant status (TAS). Furthermore, the anti-inflammatory properties of WS-5 were examined using lipopolysaccharide-stimulated BV-2 microglial cells. WS-5 significantly inhibited the lipopolysaccharide–induced production of nitric oxide and two proinflammatory cytokines, TNF-*α* and IL-6. The memory impairment in mice with A*β*-induced AD was studied using the Morris water maze and passive avoidance test. Immunohistochemistry was performed to monitor pathological changes in the hippocampus and cortex region of the mouse brain. The animal study showed that WS-5 (250 mg/kg) treatment improved learning and suppressed memory impairment as well as reduced A*β* plaque accumulation in A*β*-induced AD. HPLC analysis identified the extract's active compounds that exert anti-AChE activity. In summary, our findings suggest that WS-5 could be applied as a natural product therapy with a focus on neuroinflammation-related neurodegenerative disorders.

## 1. Introduction

Since many countries underwent the demographic transition to an aging population in recent years, the occurrence of neurodegenerative diseases has increased, and finding a treatment or cure has become the objective for many researchers worldwide [1]. Alzheimer's disease (AD) is an age-associated, irreversible, progressive neurodegenerative disease characterized by severe memory loss, unusual behavior, personality changes, and a decline in cognitive function. It is regarded as the pandemic of the 21^st^ century, imposing enormous social and economic burdens on patients and their families [2].

Several biochemical and pathological mechanisms contribute to AD. They include an imbalance between the production rate of *β*-amyloid (A*β*) and its clearance, resulting in the accumulation of A*β* by forming senile plaques [3]. Based on these observations, several antiamyloid and cholinesterase inhibitors are being investigated as disease modifying agents. Natural compounds are the major molecular structural resources for drug discovery due to features of structural diversity, thus, the potential use of natural products has attracted as extensive concern [4]. Several traditional medicinal plants and their phytochemical constituents have shown promising pharmacological activities for the treatment of AD.

WS-5, a 45% ethanol extract of a mixture of three traditional medicinal plant materials, is prepared using the rhizome of* Curcuma longa* L. (turmeric), the fruits of* Chaenomeles sinensis* Koehne (Chinese quince), and the dried rhizome of* Zingiber officinale* Roscoe (ginger) at a ratio of 2:1.5:1. Ginger is widely applied around the world as an ingredient in Ayurvedic medicine and a well-known traditional herbal medicine for the treatment of asthma, cough, rheumatism, constipation, and diabetes [5]. Previous reports have demonstrated that ginger exerts anti-inflammatory, antiulcerogenic, anti-AD, antioxidative, and neuroprotective activities in the central nervous system (CNS) [6–8]. The fruits of* Chaenomeles sinensis*, a member of the Rosaceae family, have been used as a traditional medicine in Korea, Japan, and China. Our previous report revealed that an ethanol extract of the fruits of* Chaenomeles sinensis* inhibited acetylcholinesterase (AChE) activity and reduced the secretion level of A*β*40/42 as a major strategy for preventing AD [9]. Further reports revealed that it has an antioxidant effect on* Caenorhabditis elegans*, an antidiabetic activity, an anti-inflammatory activity, and an inhibitory effect on influenza A virus [10–13].* Curcuma longa* has been widely used in traditional medicine and as a spice, food preservative, and coloring material [14]. Its rhizome contains curcumin, a major phenolic compound that has a therapeutic application in some disease ailments [15]. Previous reports revealed that* Curcuma longa* possesses multiple pharmacological activities, including antioxidant, antimicrobial, anti-inflammatory, antidementia, and antidiabetic activities [16, 17]. Specifically, experimental evidence has been provided that it can improve the cognitive function, decrease *β*-amyloid plaques, and delay deterioration of neurons in patients [18, 19].

Natural products along with their compounds have been tried searching for development of functional foods with improvement in memory function from natural products. Specially, we have desired to make a preparation with the ability to enhance the memory function and effect on the amyloidogenic pathway in AD. Previously we have carried out with 50% ethanolic extract of all herbs and as comparison to that we found that 45 % ethanolic extract showed good effect more than 50 % ethanolic extract. Therefore, by reviewing it we further decided to carry out and study with 45% ethanolic extract. As far as we are concerned* C. longa *L and* C. sinensis *Koehne are traditional folklore.* C. longa *L has been demonstrated to be therapeutically applied for Alzheimer's disease prevention and treatment whereas* C. sinensis *Koehne fruits have been developed as a tea and as per our investigation we have found it has also therapeutic values regarding several disease ailments. The basic concept for approaching WS-5 as a drug design was development of memory and cognition ability for the prevention, delay, treatment, and improvement of Alzheimer's disease comprising WS-5 extract and development of health functional food materials and products for the improvement of learning and memory abilities with therapeutic application in Alzheimer's disease. The desired preparation, WS-5, may be expected to synergistically exert the neuroprotective effect as well as enhance cognition via several mechanism based on findings from several reports.

Literature review suggests that this plants ameliorate the memory function and plays important role in AD hypothesis. On the other hand,* Curcuma longa* and* Zingiber officinale* have been combined with other plant extracts and shown to effectively enhance the memory abilities. So, in order to obtain more effective remedies for the development of memory function using natural products, we combined 45% ethanol extract of these two plants along with* Chaenomeles sinensis* to obtain potent therapeutic application in prevention of AD. Based on our results regarding* Chaenomeles sinensis* we found that it reduces the A*β* secretion and also attenuated learning and memory function on A*β*-induced memory impairment in AD mice. Based on the cholinergic hypothesis and oxidative stress involved in AD, the therapeutic effect of three plants in AD is main moto of this study.

The cholinergic deficit, senile plaque/A*β* peptide deposition, and oxidative stress have been identified as the three major pathogenic pathways that contribute to the progression of AD. Natural products are interesting treatment options for reducing the progress and symptoms of many different diseases, including AD. To test this assumption, we examined the pharmacological potential of WS-5 because of its effective use in both modern and traditional systems of medicine. We used the 1, 1-diphenyl-2-picrylhydrazyl (DPPH) free radical scavenging method to examine the in vitro antioxidative activity of WS-5, which could be used to reduce oxidative stress associated with neurodegenerative diseases. Some recent reports have suggested that flavonoid- and polyphenol-rich extracts may prevent memory impairments associated with age-related diseases [20].

In this study, we evaluated the effect of WS-5 on in vitro amyloidogenic activity, which was assessed as activity against A*β* aggregation, acetylcholinesterase, and oxidative stress, and on an* in vivo* Alzheimer's disease induced in mice by A*β* infarction. In addition, we identified major constituents of the WS-5 extract using high-performance liquid chromatography (HPLC) and assessed their anti-AChE activity. We found that WS-5 might be beneficial as a natural product therapy against AD.

## 2. Material and Methods

### 2.1. Reagents

Dimethyl sulfoxide (DMSO) was purchased from BioLife Solutions (Bothell, WA, USA). Protease inhibitor (PI) was purchased from Sigma-Aldrich (St. Louis, MO, USA). Opti-MEM, Dulbecco's modified Eagle's medium (DMEM), fetal bovine serum (FBS), and penicillin/streptomycin were obtained from Capricorn Scientific (Ebsdorfergrund, Germany). An enzyme-linked immunosorbent assay (ELISA) kit for A*β*40/42 was obtained from IBL (Kunma, Japan). The ELISA kit for tumor necrosis factor alpha (TNF-*α*), interleukin (IL-1*β*), and interleukin (IL-6) was purchased from R&D Systems (Minneapolis, MN, USA). The AChE assay kit and the Randox Total Antioxidant Status (TAS) kit were purchased from BioVision (Milpitas, CA, USA) and Randox (Crumlin, UK). Cell viability assay reagent (CCK-8) was purchased from Dogenbio Co. Ltd. (Seoul, Korea). Mouse anti-A*β*_17-26_ monoclonal antibody (4G8) was purchased from BioLegend (San Diego, CA, USA). A*β*_1-42_ was purchased from American Peptide Company, Inc. (Sunnyvale, CA, USA). Curcumin, 6-shogaol and protocatechuic acid were purchased from Sigma (St. Louis, MO, USA). All other reagents were of highest purity grade.

### 2.2. Sample Extraction

**Figure 1:**
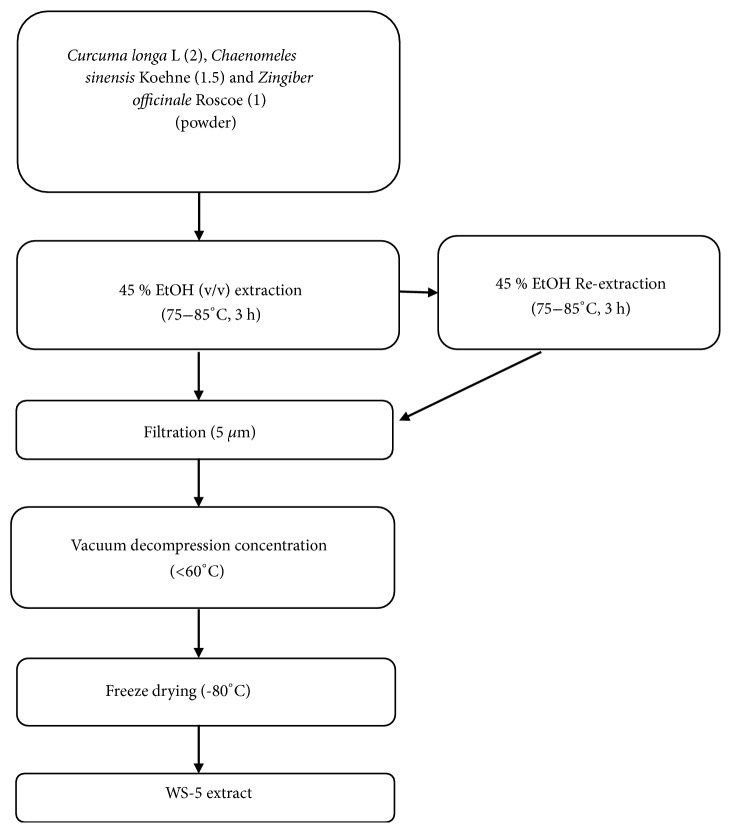
Flow chart of extraction method for preparing WS-5.

The fresh rhizomes of* C. longa *L,* Z. officinale *Roscoe, and fruits of* C. sinensis *Koehne and red ginseng were purchased from HeungIl Pharmaceutical Co. (Seoul, Korea) and were identified by one of the authors (Dr. Dae Keun Kim). The herbarium has been prepared and a voucher specimen (WSU-18-002) has been deposited in College of Pharmacy, Woosuk University, South Korea (Figure S1). The extracts were prepared using plant materials derived from the rhizome of* C. longa *L., the fruits of* C. sinensis *Koehne, and the dried rhizome of* Z. officinale *Roscoe at a ratio of 2:1.5:1 based on weight (w/w/w). The ratio of the extract, based on the weight of raw material, to the volume of 45% ethanol was 1:10 (v/w). The mixture was extracted 2 times by refluxing, at 75–85°C for 3 h. The mixture was filtered with 5 *μ*m filter paper to obtain the extract. The extract was concentrated under reduced pressure at ~60°C and then dried in a vacuum dryer at ~60°C for 18 h. It was then crushed in a crusher and wrapped with aluminum foil for subsequent experiments. The total extract yield was 3.09 kg. The final preparation was labeled as 'WS-5' [21]. Korean red ginseng (RG), steamed* Panax ginseng* C.A. Meyer, was extracted by the same extraction procedure. Briefly, RG was extracted 2 times in 10 volumes of 45% ethanol by refluxing, at 75–85°C for 3 h and filtered with 5 *μ*m filter paper. The extract was concentrated under reduced pressure at ~60°C and then dried in a vacuum dryer at ~60°C for 18 h. Finally it was crushed in a crusher and wrapped with aluminum foil for subsequent experiments and labeled as RG. Thus, RG was used as a positive control in the animal experiment. The flowchart of the extraction method is shown in Figure 1.

### 2.3. Cell Culture and Treatment

The BV-2 microglial cell line was obtained from Dr. Jin Tae Hong (Chungbuk University). The cells were plated in 75 mm tissue culture flasks and cultured in DMEM supplemented with 10% FBS and 1% penicillin-streptomycin at 37°C in a humidified atmosphere with 5% CO_2_. Cells of the flask culture were seeded in a 6-well plate when they reached almost 70% confluence.

### 2.4. Measurement of Cell Viability (CCK-8 Assay)

The Cell Counting Kit-8 (CCK-8) assay measures cell viability by the reaction of water-soluble tetrazolium salt with the dehydrogenase of living cells which produces orange-colored water-soluble formazan. BV-2 cells were cultured in a 96-well plate and incubated overnight. Then, the medium was removed and cells were washed with PBS followed by WS-5 treatment at various concentrations. On the next day, 10 *μ*l of cell viability assay reagent was added to all wells followed by 1 h incubation. The plate was shaken gently before measuring absorbance at 450 nm using a microplate reader (Bio-Rad, Hercules, CA, USA).

### 2.5. Antioxidant Assay

The antioxidant activity of WS-5 was determined using DPPH as a stable free radical. The ability of the plant extracts to scavenge DPPH and convert it into 1, 1-diphenyl-2-picrylhydrazine was determined. Briefly, WS-5 extract at concentrations of 1, 10, 50, and 100 *μ*g/ml was mixed with 247.5 *μ*l of DPPH solution (10 *μ*M in ethanol) and incubated at room temperature for 20 min in dark. The optical density values of the mixtures were measured at 517 nm with a spectrophotometer (Tecan, Mannedorf, Switzerland). Vitamin C was used as positive control.

### 2.6. Total Antioxidant Status (TAS) Assay

The TAS kit (Randox, Crumlin, UK) was used to determine the quantitative in vitro total antioxidant status of the WS-5 extract. A 96-well plate was loaded with 4 *μ*l per well of each test sample at the final concentration of 1, 10, 50, and 100 *μ*g/ml; 200 *μ*l of Reagent R_2_ (chromogen) was added for a 2 min reaction at 37°C. The initial absorbance A_1_ at 600 nm was measured using a spectrophotometer (Tecan, Mannedorf, Switzerland); 40 *μ*l of reagent R3 (substrate) was immediately added and the A_2_ value was measured after exactly 3 min. The absorbance of distilled water (DW) was the blank control, DMSO was the negative control, and vitamin C was the positive control. TAS was determined following the manufacturer's protocol.

### 2.7. Anticholinesterase Activity

AChE inhibitory activity was determined by the AChE colorimetric assay (BioVision, CA, USA). An aliquot of 30 *μ*l of the WS-5 extract and compounds were added to a 96-well plate at a concentration of (1, 10, 50, 100 *μ*g/ml) and (10, 50, 100 *μ*M); 1 *μ*M of tacrine was used as positive control. The reaction mixture (50 *μ*l), followed by 10 *μ*l of diluted AChE, was added. The absorbance was measured at 570 nm using an ELISA reader (Bio-Rad, California, USA).

### 2.8. Transmission Electron Microscopy (TEM) Assay

An assay to monitor A*β* oligomer and fibril formation using TEM images was performed. Briefly, 20 *μ*M of A*β*_1-42_ (10 *μ*l) was incubated at 37°C along with WS-5 (10 *μ*l of 10 *μ*g/ml) for 0, 5, and 24 h. The samples were applied to a formvar-carbon coated grid (400 mesh copper grids, Electron Microscopy Sciences, Hatfield, PA, USA), dried, and stained with 1% uranyl acetate solution for 1 min. The stained samples were air dried. Then, the sample grids were analyzed by TEM using images obtained with an H-7650 FEI G2 Spirit Twin Microscope 80 KV (Hitachi, Tokyo, Japan) at magnifications of 50,000× and 100,000×.

### 2.9. Determination of Nitric Oxide (NO) Production

The inhibitory effect of WS-5 on lipopolysaccharide (LPS)-stimulated NO release was measured by the Griess reaction. Briefly, BV-2 microglial cells were seeded on a 96-well plate and treated with LPS with or without WS-5 extract for 24 h. The culture supernatant was collected and measured using the Griess reagent (0.1% N-(1-naphthyl)-ethylenediamine dihydrochloride and 1% sulfanilamide in 5% phosphoric acid); 100 *μ*l of sample was mixed with the same volume of Griess reagent and incubated for 10 min at room temperature in the dark. The absorbance of each well was measured at 540 nm using a microplate reader (Tecan, Mannedorf, Switzerland).

### 2.10. Measurement of TNF-*α* and IL-6 Production

ELISA kits were used to determine the levels of proinflammatory cytokines, TNF-*α* and IL-6 (R&D Systems Inc., Minneapolis, MN, USA). Briefly, BV-2 cells were seeded on a 6-well plate in DMEM and incubated for 24 h. Cells were stimulated with LPS in the presence or absence of WS-5. After 24 h incubation, the culture supernatant was used for measuring the level of TNF-*α* and IL-6 according to the manufacturer's instructions.

### 2.11. Animals

All animal procedures were in accordance with and approved by the Animal Care Committee of the Woosuk University. ICR male mice, 3 weeks of age with an average weight of 20–25 g, were obtained from Damul Science (Daejeon, Korea). They were housed comfortably in a group of ten per cage with a metal frame lid on its top. The mice were kept in standard environmental condition (at 23–25°C temperature, 55–65% relative humidity, and 12 h light/12 h dark cycle) for 3 weeks for acclimation with* ad libitum* access to rodent food and water.

### 2.12. Treatment and Experimental Protocol

All mice were divided into four groups (n=10 in each group) as follows:

Group I saline group: mice were orally treated with DW, which served as vehicle control group after intracerebroventricular injection (ICV) with normal saline (5 *μ*l/mice), once daily for 14 days.

Group II Alzheimer group (A*β*^+^): mice were orally treated with DW once daily for a period of 14 days after the intracerebroventricular injection (ICV) with amyloid-*β* (5 *μ*l/mice).

Group III A*β*^+^ + RG: mice were treated orally with red ginseng at a dose of 100 mg/kg for a period of 14 days after the intracerebroventricular injection (ICV) with amyloid-*β* (5 *μ*l/mice).

Group IV A*β*^+^ + WS-5: mice were treated orally with WS-5 at a dose of 250 mg/kg for a period of 14 days after the intracerebroventricular injection (ICV) with amyloid-*β* (5 *μ*l/mice).

**Figure 2:**
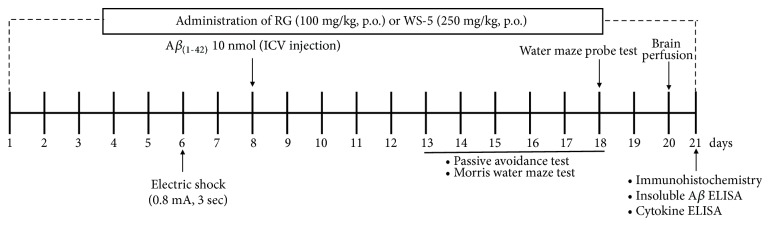
Timeline of the in vivo experiment including the extract treatment regimens. Mice were treated with WS-5 or RG extract for 20 days until mice perfusion. A*β*_1-42_ (10 nM; ICV injection) was administered on the 8^th^ day followed by the step-through test and the water maze test. On the 6^th^ day after A*β*_1-42_ administration, the passive avoidance test was conducted. After brain perfusion, biopsy was performed and the recovered brain tissue samples were analyzed by ELISA.

All mice were treated orally with assigned substances for a period of 15 days after the intracerebroventricular injection of A*β* peptide or saline which underwent behavioral assessment. After the behavioral test, mice were sacrificed and brain was taken for biochemical studies. The timeline for in vivo animal experiment is shown in Figure 2.

### 2.13. Intracerebroventricular (ICV) Injection of Amyloid-*β* Protein (*Aβ*_1-42_)

All the surgical equipments were autoclaved before surgery. Briefly, mice were anesthetized by intraperitoneal injection with Avertin and mounted in a stereotaxic frame. A*β*_1-42_ peptides (10 nM) at a volume of 5 *μ*l were delivered by ICV injection, with stereotaxic coordinates, 0.22 mm posterior to bregma, 1.0 mm lateral to the sagittal suture, and 2.5 mm depth from the skull surface using a Hamilton microsyringe (Hamilton, Reno, USA) fitted with a 26 G stainless-steel needle (Paxinos and Franklin, 2012). The stereotaxic injection was performed at a speed of 1 *μ*l/min with an automatic syringe pump (Sercrim, Labtech, South Korea). The vehicle-operated mice were injected with the same volume of sterile saline. After surgery, mice were kept warm in house caged and monitored until full recovery.

### 2.14. Behavioral Assessment

#### 2.14.1. Passive Avoidance Test

The passive avoidance test is traditionally used to assess short-term and long-term memory in rodent models of CNS disorders [22]. Passive avoidance task was determined using the step-through method (Ugo Basile, Gemonio, Italy) which consisted of a tilted floor box divided into two compartments by a sliding door and a control unit with an incorporated scrambler shocker. On the training trial, the mice were placed in the light compartment and, after 30 s, the sliding door was opened. When the mice moved into the dark compartment, they received a foot shock with 0.8 mA electricity for 3 s through a stainless-steel grid positioned in dark compartment. On the 6^th^ day, after the A*β*_1-42_ infusion, the same test procedure was performed without foot shock. When the mice did not enter the dark compartment, the latency time was recorded for 300 s.

#### 2.14.2. Morris Water Maze Test

The water maze apparatus consisted of a circular pool (height, 30 cm; diameter, 100 cm) filled with water and maintained at ~20–22°C. An escape platform (height, 35.5 cm; diameter, 4.5 cm) was inserted in the center of the quadrant of the pool every day except on the last day of the experiment. Spatial learning and memory performance were tested for 5 consecutive days beginning at 6^th^ day after the infusion of A*β*_1-42_ peptide. Each mouse was trained to find the hidden platform whereas escape latency and swimming pattern were recorded. A test trial was conducted on the 6^th^ day to assess memory consolidation when the platform was removed and mice were allowed to swim freely. Target crossing, escape latency, and distance traveled across the hidden platform were monitored for each mouse by a video camera linked to the center of the pool and connected to a SMART-CS program (Panlab, Barcelona, Spain).

### 2.15. Immunohistochemistry

In preparation for the immunohistochemistry analysis, mice brains were perfused with Tris-buffered saline (pH 7.6) followed by 4% paraformaldehyde in PBS pH 7.4 at 4°C. Brains were subsequently postfixed in the same fixative. The fixed tissues were embedded in a cryostat cutting medium and coronal sections of 20 *μ*m thickness were cut on a cryostat. Immunohistochemistry analysis was conducted following a standard avidin-biotin complex method. Briefly, specimens were blocked with 5% normal goat serum, 2% BSA and 3% FBS. To visualize the formation of the antigen-Ab complex, sections were incubated overnight with the 4G8 anti-A*β* antibody (Biosource, Camarillo, USA) at a dilution 1:400. Color development was done using 3, 3′-diaminobenzidine.

### 2.16. Mouse Brain A*β*/Cytokine ELISA

Insoluble A*β* ELISA was performed in a 96-well plate which permits a high throughput test. Mouse brains were homogenized with PI and 88% formic acid. The mixture was centrifuged at 100,000 ×*g*. The supernatant was added to each well and incubated at 4°C for 16 h. Horseradish peroxidase-conjugated A*β* monoclonal antibody was added to each well, incubated at 4°C for 1 h, and washed 7 times with washing buffer. 3,3′,5,5′-Tetramethylbenzidine (TMB) was added to each well and incubated for 30 min after washing 9 times. The reaction was stopped by adding 100 *μ*l stop solution and absorbance at 450 nm was measured using an ELISA reader (Bio-Rad). Proinflammatory cytokine levels (IL-1*β*) were determined using the commercially available ELISA kit from R & D Systems (Minneapolis, MN).

### 2.17. Quantification of WS-5 by HPLC Analysis

Marker compounds of WS-5 were detected using the HPLC system Shiseido with photodiode array detector. The elution profile was monitored at 254 nm using a column temperature of 40°C. Chromatography was performed using a Capcell Pak C18 MG II (250 Χ 4.6 mm, 5 *μ*M; Shiseido Co., Ltd., Tokyo, Japan). The mobile phase was composed of 0.1% trifluoroacetic acid in water (solvent A) and 0.1% trifluoroacetic acid in acetonitrile (solvent B). The gradient program was 0-10 min, 100% of solvent A; 10-13 min, 90% of solvent A; 13-20 min, 60% of solvent A; 20-25 min, 60% of solvent A; 25-35 min, 50% of solvent A; 35-40 min, 50% of solvent A; 40-45 min, 55% of solvent A; 45-55 min, 30% of solvent A; 55-60 min, 30% of solvent A with a flow rate of 1 ml/min and the injection volume was 10 *μ*l.

### 2.18. Statistical Analysis

All data are presented as the mean ± S.E.M. Statistical evaluation between experimental groups was performed using one-way analysis of variance (ANOVA) followed by Tukey's post hoc test using Graph Pad Prism 5.01 (Graph Pad Software, Inc., La Jolla, CA, USA).* p*<0.05 was considered as statistically significant. Each experiment was performed in triplicate.

## 3. Results

### 3.1. WS-5 Inhibited AChE

**Figure 3:**
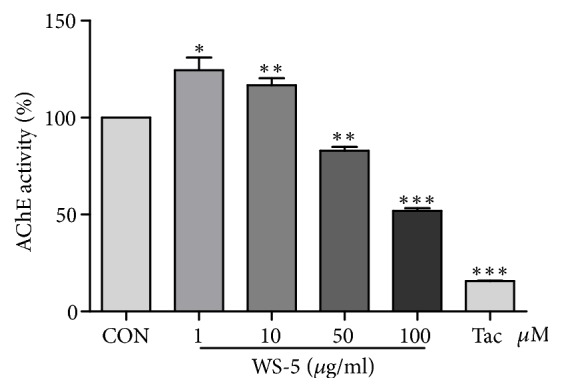
AChE inhibitory activity of WS-5. WS-5 was prepared at final concentrations of 1, 10, 50, and 100 *μ*g/ml with AChE assay buffer. The assay was performed by mixing diluted sample with AChE substrate (5 mM), choline oxidase enzyme, and AChE probe and incubating at 37°C for 20 min. Inhibition of AChE was measured using the AChE assay kit, with 1 *μ*M of tacrine as positive control. Absorbance was measured at wavelength of 570 nm using a microplate reader (Bio-Rad, Hercules, CA, USA). Results are expressed as the means ± SEM of three independent experiments (^*∗*^*p* < 0.05, ^*∗∗*^*p* < 0.01, ^*∗∗∗*^*p* < 0.001* vs*. control).

Many reports have demonstrated that AChE inhibitors from plant materials could be potentially used as therapeutic agents for neurodegenerative diseases like AD [23]. In our study, WS-5 at various concentrations along with tacrine as positive control was investigated for AChE inhibitory activity. WS-5 significantly inhibited the AChE activity in a dose-dependent manner. Specifically, WS-5 decreased AChE activity by 51.98% at 100 *μ*g/ml with an IC_50_ value of 84.10 *μ*g/ml (Figure 3).

### 3.2. WS-5 Showed Potent Antioxidant Activity

**Figure 4:**
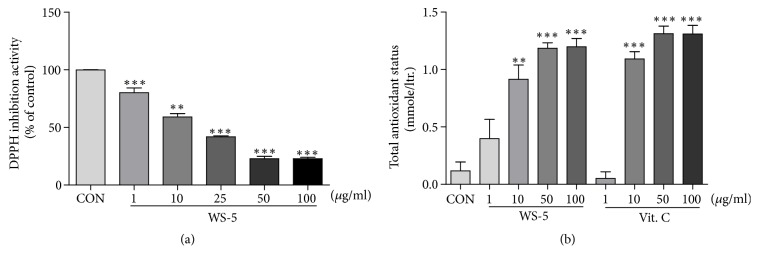
DPPH radical scavenging activity and TAS of WS-5. Inhibition of DPPH was measured using the radical scavenging assay, with vitamin C as positive control, along with the TAS assay. Results are expressed as the means ± SEM of three independent experiments (^*∗*^*p* < 0.05, ^*∗∗*^*p* < 0.01, ^*∗∗∗*^*p* < 0.001* vs*. control).

Extensive scientific evidence suggests that oxidative stress is involved in age-related neurodegenerative diseases. In many studies, the potential of reducing neuronal death by antioxidants has been explored [24]. Thus, the antioxidant activity of the WS-5 extract was evaluated by measuring its radical scavenging activity using the DPPH and TAS assay. WS-5 showed high antioxidant activity, as indicated by the dose-dependent inhibition of quenching the free radicals. The IC_50_ value of WS-5 for the DPPH radical scavenging activity was 28.32 *μ*g/ml. The antioxidative activity was confirmed by the TAS assay, which showed a high amount of oxidant content in the extract as compared to that in the vitamin C positive control (Figures 4(a) and 4(b)).

### 3.3. WS-5 Inhibits *Aβ*_1–42_ Aggregate Formation

**Figure 5:**
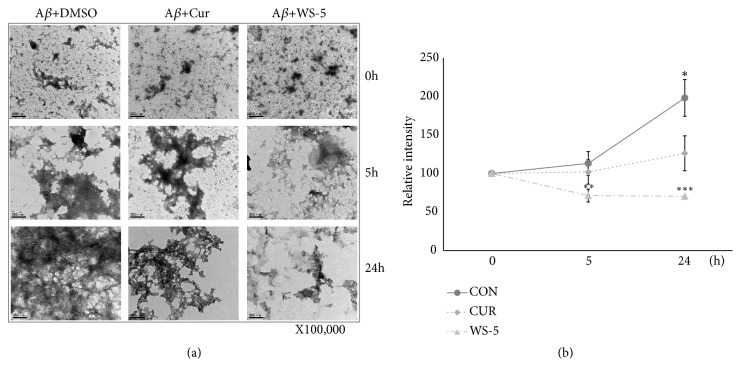
WS-5 inhibits A*β* aggregation. In preparation for TEM analysis, 10 *μ*g/ml of WS-5 was coincubated with 20 *μ*M A*β*_1-42_ for 0, 5, and 24 h. TEM images of control, positive control, and WS-5 treated samples are shown. Images were quantified using Image J software. Scale bar indicates 200 nm (at 100,000× magnification). Values are presented as the means ± SEM of three independent experiments (^*∗*^*p* < 0.05, ^*∗∗*^*p* < 0.01, ^*∗∗∗*^*p* < 0.001* vs*. control).

A*β* aggregation is a complex, multistep process that may include the merging of oligomers to form the fibril complex. However, the precise aggregation pathway is not yet completely understood. We investigated the effects of WS-5 on A*β*_1–42_ aggregation with or without WS-5 using the TEM method. The samples, along with a positive control, were incubated for 0, 5, and 24 h. In the 5 h test, WS-5 significantly inhibited A*β*_1–42_ aggregate formation as compared to the control (DMSO) and the curcumin sample (positive control). This result was confirmed by the 24 h test. The morphology analysis indicated that the WS-5 extract significantly inhibited aggregate formation in a time-dependent manner (Figures 5(a) and 5(b)).

### 3.4. Cytotoxic Effect of WS-5 on BV-2 Cells

**Figure 6:**
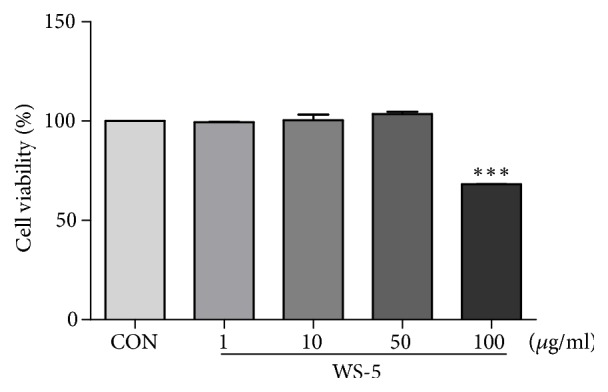
Effect of WS-5 on viability of BV-2 cells. BV-2 cells were treated with various concentration of WS-5 for 24 h and cell viability was determined using the CCK-8 reagent. Values are presented as the means ± SEM of three independent experiments (^*∗*^*p* < 0.05, ^*∗∗*^*p* < 0.01, ^*∗∗∗*^*p* < 0.001* vs*. control).

The cytotoxic effect of different concentrations of WS-5 (1, 10, 50, 100 *μ*g/ml) was evaluated on BV-2 cells. The cells were treated with WS-5 for 24 h and their viability was determined using a cytotoxicity assay reagent. The extract showed a cytotoxic effect of 31% at the concentration of 100 *μ*g/ml (Figure 6).

### 3.5. WS-5 Inhibits NO release and Proinflammatory Cytokine Production in LPS-Induced BV2 Microglial Cells

**Figure 7:**
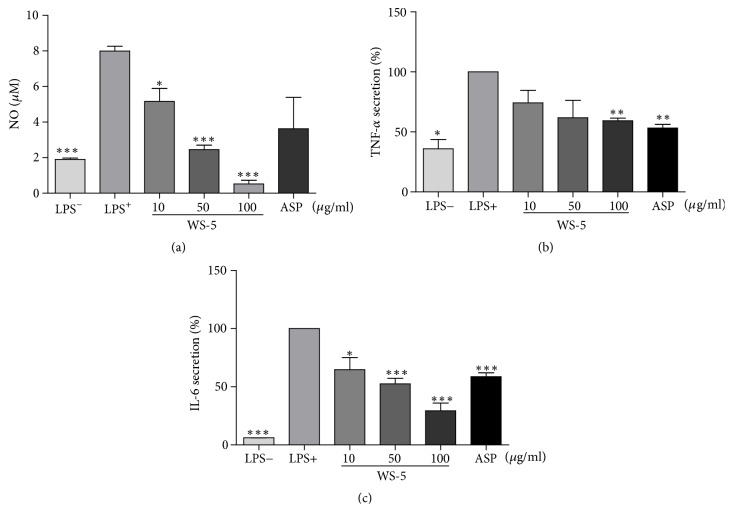
WS-5 inhibits NO release (a) and production of TNF-*α* and IL-6 (b, c) in LPS-stimulated BV-2 microglial cells. The cells were treated with or without WS-5 for 24 h. The nitrite concentration was determined using the Griess reagent; the concentrations of TNF-*α* and IL-6 in the culture supernatant were measured using a commercial ELISA kit. Values are presented as the means ± SEM of three independent experiments (^*∗*^*p* < 0.05, ^*∗∗*^*p* < 0.01, ^*∗∗∗*^*p* < 0.001* vs*. control).

WS-5 significantly decreased LPS-induced NO production in a concentration-dependent manner relative to the control treatment (Figure 7(a)). WS-5 also significantly suppressed the proinflammatory cytokines TNF-*α* and IL-6 in a concentration-dependent manner (Figures 7(b) and 7(c)), indicating that WS-5 possesses anti-inflammatory properties.

### 3.6. Effect of WS-5 on the Performance of A*β*-Induced AD Mice in Behavioral Tests

#### 3.6.1. WS-5 Ameliorates Learning and Memory Impairment in the Passive Avoidance Test

**Figure 8:**
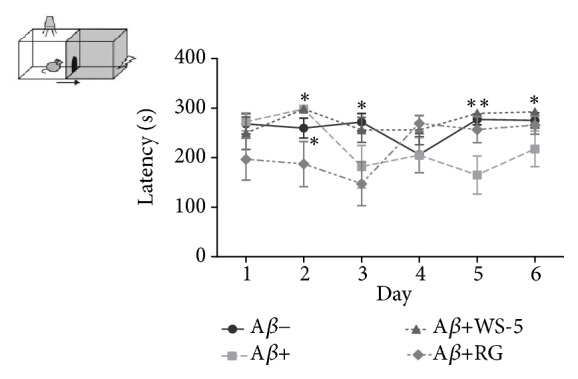
Effect of WS-5 on cognitive improvement of mice. A*β*-injected ICR mice received WS-5 extract or RG control extract by oral administration (WS-5, 250 mg/kg; RG, 100 mg/kg) for 3 weeks before memory tests were conducted. After a mouse entered the dark box, it was subjected to an electric foot shock (0.8 mA for 5 s). During the training trial, the time (latency) to enter the dark box was scored for each mouse. Then, 24 h after the training trial, a retention test was conducted in which the same animals were put into the illuminated box and again the latency to enter the dark box was recorded (^*∗*^*p* < 0.05, ^*∗∗*^*p* < 0.01* vs*. A*β*^+^ treated group).

We evaluated learning and memory capabilities by administering the passive avoidance test using the step-through method. In this test, the step-through latency was significantly increased in the mice treated with WS-5 as compared with that in the mice of the A*β*^+^ control group. The result remained consistent during the entire test period (Figure 8).

#### 3.6.2. WS-5 Improves Acquisition of Spatial Memory Deficits in the Morris Water Maze Test

**Figure 9:**
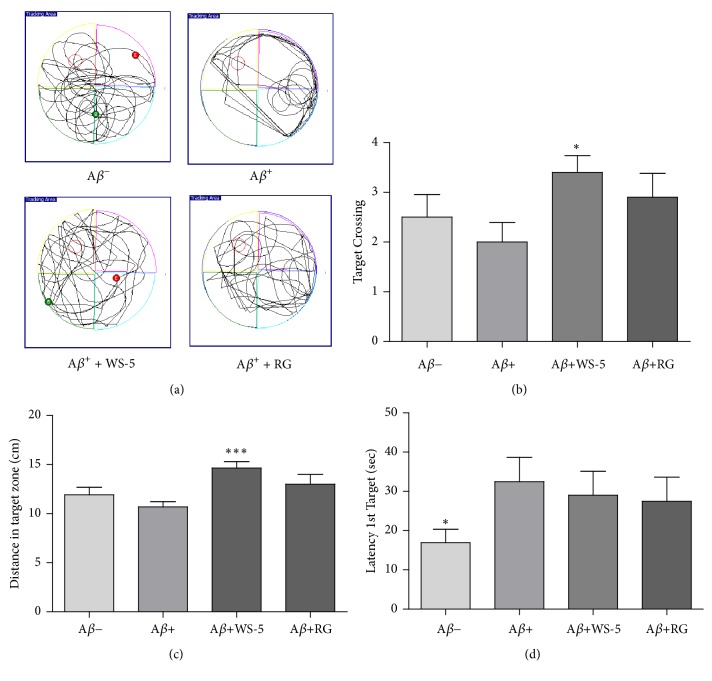
Effect of WS-5 extract on the performance of A*β*-induced mice in the water maze test. (a) Traces show the swimming pattern of each group during test trial. (b) Escape latency to find the target platform on the final day. (c) Total number of crossing the platform on the final day. (d) Distance taken to cross target zone on the final day. Data are expressed as the means ± SEM of the experiment (^*∗*^*p* < 0.05, ^*∗∗∗*^*p* < 0.001* vs*. A*β*^+^ treated group).

For monitoring spatial memory deficits, the time required to find a hidden platform was assessed by administering the Morris water maze test. The test to assess memory retention was performed on the 6^th^ day following a 5-day training trial. The swimming path of mice in the final test trial is shown in Figure 9(a). The mice in each group except the A*β*^+^ treated group showed the tendency to find and move around the platform relating to spatial learning, whereas the A*β*^+^ group used other search routes. Escape latency was shorter in the WS-5 group than that in the A*β*^+^ control group (Figure 9(d)). Moreover, the distance taken to find the platform was markedly improved in the WS-5 group as compared to that in the A*β*^+^ control group (Figure 9(c)). In addition, the crossing platform times were significantly increased in the WS-5 treated group as compared to that in the positive control group (Figure 9(b)). These results demonstrated that the WS-5 group improved the spatial memory capabilities according to the tests for escape latency and target crossing towards the hidden platform.

### 3.7. WS-5 Suppressed the Accumulation of A*β* Plaques in the Hippocampus and Cortex Region of the Mouse Brain

**Figure 10:**
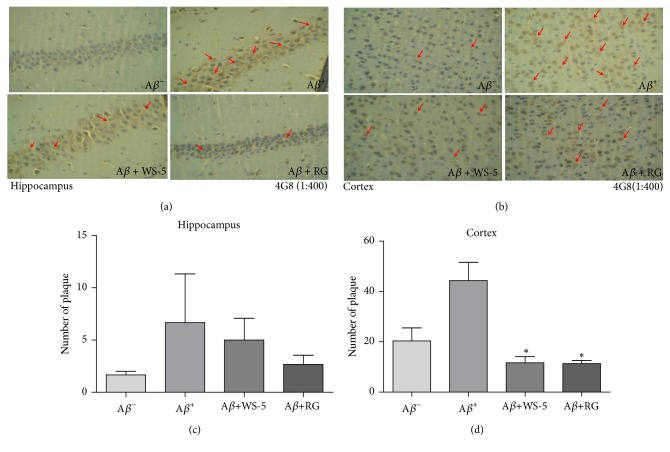
Effect of WS-5 on A*β* deposition in the cortex and hippocampus region of the mouse brain. A*β* accumulation in the brain was determined by immunohistochemistry analysis using 4G8 anti-A*β* antibody (1:400). An arrow head indicates accumulation of amyloid plaques, which is higher in the A*β*^+^ group than in the other groups. Scale bar indicates 25 *μ*m (400× magnification). Data are expressed as the means ± SEM of the experiment (^*∗*^*p* < 0.05* vs*. A*β*^+^ treated group).

Because the extracellular accumulation of A*β* in senile plaques is a pathological hallmark of AD, many preventive strategies are focused on suppressing the plaque formation and accumulation. We analyzed the pathological changes in the hippocampus and cortex region of mouse brains via immunohistochemistry as described in Materials and Methods section. The histopathological analysis using the 4G8 anti-A*β* antibody showed an advanced A*β* accumulation in the cortex and hippocampus region of the A*β*^+^ group mice. Although there was significant reduction in A*β* plaque in cortex region, in hippocampus region we could not accomplish the significant reduction. In sharp contrast, the accumulated A*β* plaque burden was remarkably reduced by the administration of WS-5 in both brain regions, according to the results in the WS-5 group mice (Figures 10(a), 10(b), 10(c) and 10(d)).

### 3.8. WS-5 Reduced the Secretion of Major Proinflammatory Cytokines and the A*β*40 Level in the Mouse Brain

**Figure 11:**
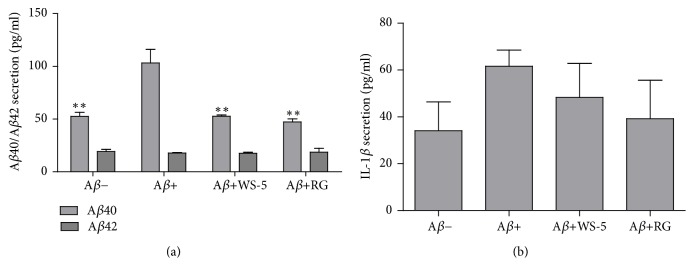
Effect of WS-5 on the levels of A*β*40/42 and proinflammatory cytokines IL-1*β* in the mouse brain. (a) Insoluble A*β*40/42 level in mouse brain. The mouse brain was homogenized with PI and 88 % formic acid and centrifuged at 30,000 rpm. The supernatant was taken for ELISA assay. (b) IL-1*β* secretion level in mouse brain. The mouse brain was homogenized with cold lysis buffer and centrifuged at 30,000 rpm. Proinflammatory cytokine level (IL-1*β*) was determined using commercially available enzyme-linked immunosorbent assay kit according to the manufacturer's instructions. Data are expressed as the means ± SEM of the experiment (^*∗*^*p* < 0.05, ^*∗∗*^*p* < 0.01* vs*. A*β*^+^ treated group).

We performed a quantitative analysis to determine the A*β*40/42 levels and the proinflammatory cytokine levels, which are increased during microglia activation caused by A*β* deposits. The quantitative analysis revealed that the WS-5 treated group had a significantly decreased A*β*40 level as compared to that of the A*β*^+^ group whereas all groups had a similar A*β*42 level (Figure 11(a)). Further, WS-5 treatment reduced the level of secreted IL-1*β* (Figure 11(b)) whereas the reduction of the level of secreted IL-6 caused by the WS-5 treatment was not significant as compared to that of the A*β*^−^ group (data not shown).

### 3.9. HPLC Analysis of WS-5

**Figure 12:**
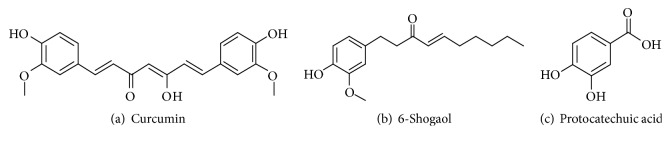
Chemical structures of three compounds: curcumin (a), 6-shogaol (b), and protocatechuic acid (c).

**Figure 13:**
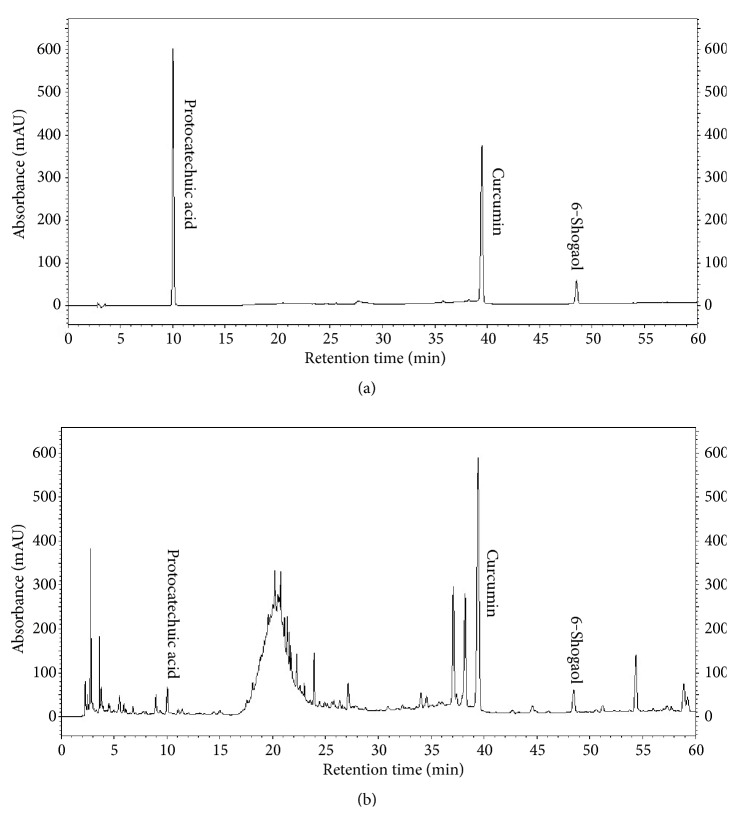
HPLC chromatogram showing the quantification of WS-5 with the marker compounds. Chromatogram: (a) three standard compounds and (b) WS-5 extract.

To ensure standardization of WS-5, we performed HPLC to detect and quantify the marker compounds of the extract. Three compounds, curcumin, 6-shogaol, and protocatechuic acid, were detected in the WS-5 extract using the validated retention times of standardized compound preparation for their identification (Figure 12). Along with that 6-gingerol was also detected in WS-5 using validated retention times (Figures S2, S3 and Table S1). The coefficient of determination (r^2^) and linearity of each compound were determined using five different concentrations (Table 1). In WS-5, the content of curcumin (7.123 ± 0.22 mg/g) was higher than that of 6-shogaol (1.896 ± 0.001 mg/g), or protocatechuic acid (0.432 ± 0.001 mg/g). Representative HPLC chromatograms of the WS-5 extract and the standardized compounds are shown in Figures 13(a) and 13(b).

### 3.10. Assessment of Anti-AChE Activity of WS-5 and Its Active Compounds

**Figure 14:**
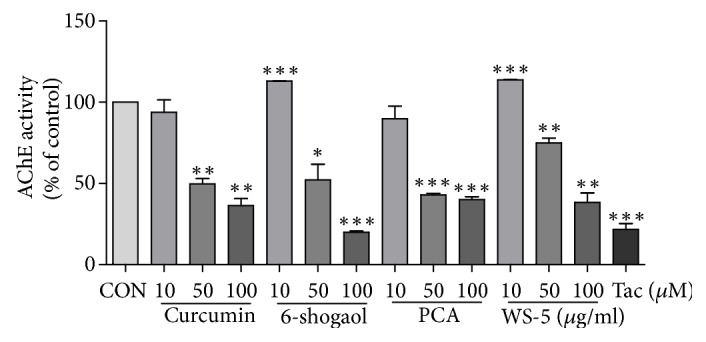
Measurement of antiacetylcholinesterase activity (AChE) of WS-5 (10, 50,and 100 *μ*g/ml), its marker compounds (10, 50, and 100 *μ*M), and tacrine (1 *μ*M) as the positive control. PCA, protocatechuic acid. Results are expressed as the means ± SEM of three independent experiments (^*∗*^*p* < 0.05, ^*∗∗*^*p* < 0.01, ^*∗∗∗*^*p* < 0.001* vs*. control).

Many studies have focused on natural product compounds from plants as a source for potential therapeutics for the treatment of AD. In this study, we focused on assessing the anti-AChE activity of marker compounds found in WS-5 as an experimental approach to identify novel AChE inhibitors. The results of the AChE inhibition test using WS-5 and its marker compounds as well as a positive control, tacrine, are provided in Figure 14. The compounds and the WS-5 extract exerted anti-AChE activity in a dose-dependent manner. The half-maximal inhibitory concentration (IC_50_) values of WS-5 and its marker compounds are presented in Table 2. WS-5, curcumin, 6-shogaol, and protocatechuic acid had an IC_50_ of 84.10 *μ*g/ml, 25.52 *μ*g/ml, 17.92 *μ*g/ml, and 10.42 *μ*g/ml, respectively. Among these, 6-shogaol possessed the strongest inhibitory activity, identifying it as a potent AChE inhibitor.

## 4. Discussion

In this study, we found that WS-5, an ethanol extract derived from* C. longa* L.,* C. sinensis* Koehne, and* Z. officinale* Roscoe, affected in vitro amyloidogenic activities as a strong antioxidant with inhibitory activity against AChE and A*β* aggregation. Furthermore, in vivo experiments in mice with A*β*-induced AD demonstrated that WS-5 possesses effective cognition enhancing activity. We also found that WS-5 suppressed A*β* plaque formation in the A*β*-induced AD mice. Moreover, we discovered that the three WS-5 marker compounds possessed anti-AChE activity that may have contributed to the anti-AChE activity of the WS-5 extract.

AD is a severe and chronic disorder of the brain and a leading cause of death among the elderly [25]. Among several degenerative features that have been identified, the oxidative imbalance and cholinergic dysfunction are considered as the major contributing factors in the pathogenesis of AD [26]. Therefore, a drug candidate that exerts protection and inhibition against oxidative stress and cholinergic dysfunction would be considered as an effective drug candidate for the treatment of AD. The exploration of novel drug candidates has shown that natural products such as plant extracts and plant derived compounds have a great potential as neuroprotective agents. Several reports have demonstrated that curcumin derivatives inhibit A*β* aggregation and induce the formation of low-molecular size A*β* species [27].

The future aim of our work is to develop functional foods comprising natural plant extracts that improve the cognitive abilities in the prevention or treatment of AD. To date, there are no reports on using* C. longa *L. and* C. sinensis *Koehne as a functional food in Korea. Therefore, we investigated the underlying mechanism of the activities associated with the combined plant extract that could be an interesting source for the development of novel functional foods. Each of the three plants has been reported to possess anti-AChE [9], anti-inflammatory [28], and neuroprotective [29] activities that are considered to be key properties of AD drugs. Based on those reports, we decided to further investigate the potential mechanism of action of a preparation derived from a combination of these three plants. Several lines of evidence have suggested that cholinesterase inhibitors approved by the Food and Drug Administration (FDA) are agents for AD treatment [30]. Current research focuses on the development of new therapeutic agents from natural products. Here, we investigated the effect of WS-5 on AChE activity, reaching half-maximal inhibition at a concentration of 100 *μ*g/ml extract.

Antioxidants, which are considered helpful in treating AD, scavenge free radicals and reactive oxygen species (ROS), reducing oxidative stress and associated cellular damage [31]. Our study demonstrated that WS-5 extract has a high antioxidant activity which could be effective in reducing the cellular damage [32]. A*β* aggregation is a critical step in AD that induces neurotoxic species and their deposition in the brain, leading to neuroinflammation and neurite degeneration [3]. The therapeutic strategies for AD are mainly focused on inhibiting A*β* neurotoxicity by blocking the formation of various forms of A*β* aggregates [33]. Curcumin, which is the active constituent of* C. longa*, has been shown to block aggregation and fibril formation in vitro and in vivo [34]. Consistent with previous reports, our results also indicated that WS-5 inhibited A*β* aggregation and possess high antioxidant activity. Recently, it was reported that high antioxidant activity interferes with self-assembly of *β*-sheets, indicating that antioxidant activity may also inhibit A*β* aggregation [35, 36].

Activation of microglial cells results in the production of major proinflammatory cytokines, TNF-*α* and IL-6, during CNS inflammation, which is typically associated with neurodegenerative diseases including AD [37]. TNF-*α* acts as an autocrine mediator causing prolonged activation of microglial cells [38]. However, overproduction of proinflammatory cytokines by activated microglial cells affects neuronal cells. Therefore, the reduction of these cytokines is a key target for inducing protection against neuroinflammation [39]. Accordingly, we investigated whether WS-5 inhibits the production of the proinflammatory cytokines in BV-2 microglial cells. Interestingly, our result showed that WS-5 significantly inhibited the production of proinflammatory cytokines in a dose-dependent manner. Thus, our data indicated that the strong anti-inflammatory activity of WS-5 inhibited activated microglia.

A recent report has suggested that* C. longa* L. helps to prevent memory dysfunction in scopolamine induced memory impairment in mice [40]. An* in vivo* experiment also revealed that* C. sinensis* extract improved trimethyltin (TMT)-induced deficits in learning and memory in mice, indicating its effect on attenuating TMT-induced brain disorder [41]. To our knowledge, this is the first report that WS-5, a combined ethanol extract of* C. longa* L.,* C*.* sinensis *Koehne, and* Z*.* officinale *Roscoe, has preventive effects on A*β*-induced AD in mice. Mathew et al. [7] reported that the value of IC_50_ of ginger extract against acetylcholinesterase is 41 *μ*g/ml. Karam et al. [30] suggested that oral administration of 216 mg/kg of ginger extract significantly inhibited AChE in rats. Our study shows that WS-5 had IC_50_ value of 84 *μ*g/ml for inhibition of AChE (Figure 3). Furthermore, we determined the cell viability on APPswe cell line (neuroblastoma cell which expresses APPswe mutation) in vitro (data not shown) which showed WS-5 possessed cytotoxic effect of 38 % at the concentration of 100 *μ*g/ml similar to BV-2 cell (Figure 6). According to this result, the doses of WS-5 and RG for treatment to mice were finally determined as 250 mg/kg and 100 mg/kg. Our in vivo experiment demonstrated that oral administration of WS-5 (250 mg/kg) ameliorated the A*β*-induced memory impairment assessed by behavioral tasks, i.e., passive avoidance and water maze test (Figures 8 and 9). However, using the Y-maze test, we did not observe that WS-5 reversed the deficit in willingness to explore a new environment and the spatial working memory (data not shown). Importantly, A*β* accumulation, which is considered to be a major cause of AD [42], was reduced by the administration of WS-5 extract in cortex region of mouse brain (Figures 10(a) and 10(c)). Immunohistochemistry result showed higher number of A*β* plaques in cortex region than in hippocampus which might be the reason that the significant reduction has been shown in cortex region only. Long-term memory is also associated with the hippocampus which is interconnected with the cerebral cortex region. The role of cortex region along with basal ganglia also highlights the spatial learning and memory ability from motor behavior and imaging studies [43]. Thus, our results demonstrated that WS-5 treated group reduced the plaque burden in cortex region significantly which may be associated with memory deficits although not significant reduction in hippocampus (Figures 10(b) and 10(d)). In the present study, there was increase in the cytokine level of IL-1*β* in the A*β* induced AD mice compared to vehicle and WS-5 operated mice (Figure 11(b)). Elevated levels of these cytokines clearly indicate the ongoing chronic inflammatory cycle in the AD mice. Therefore, the memory-enhancing effect of WS-5 may occur partly via decreased A*β* secretion, inhibiting cytokines level (IL-*β*) and reduction in A*β* plaque burden by enhancing the AD's cascade hypothesis.

Bioactive compounds are known to have pharmacological and toxicological effects contributing their effect on plants. We tested for three major compounds: curcumin, 6-shogaol, and protocatechuic acid from WS-5 to validate their content in the WS-5 extract. We found that WS-5 had a high content of curcumin as compared to that of the other two major compounds (Table 1). Further, we tested these compounds for anti-AChE activity and compared their activity with that of the WS-5 extract. We found that these compounds exerted strong AChE inhibitory activities indicated by their low IC_50_ values, curcumin (69.27 *μ*M), 6-shogaol (64.85 *μ*M), and protocatechuic acid (67.58 *μ*M) which may have contributed to the anti-AChE activity of WS-5 (Table 2).

**Figure 15:**
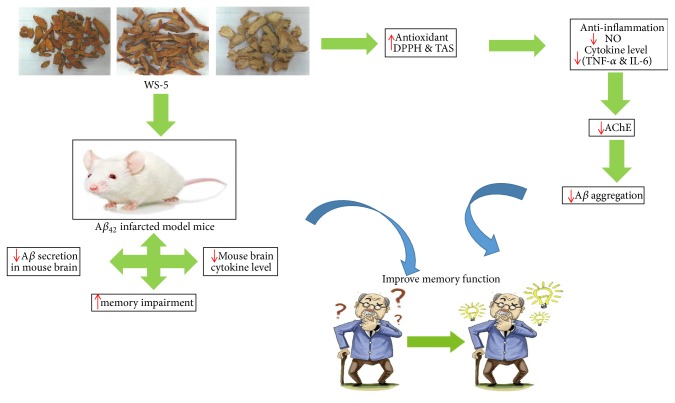
Schematic diagram showing possible underlying mechanism of WS-5.

Many studies have suggested that uses of antioxidant and anti-inflammatory agents such as melatonin, curcumin, blueberry, and EGB761 (the standardized extract of Gingko biloba) effectively alleviate the symptoms of AD models and/or AD patients in independent laboratory or clinical experiments and are suggested as promising strategies for treatment of AD [44–46]. The possible underlying mechanism of WS-5 could be that WS-5 affects in vitro amyloidogenic activities as a strong antioxidant with inhibitory activity against AChE and A*β* aggregation whereas alleviates the in vivo A*β*-induced memory impairment in passive avoidance and Morris water maze tests (Figure 15). These results support the rationale that the complex of organic materials, such as WS-5, containing cholinergic, antioxidative and anti-inflammatory activities together, may be as promising as drugs for the treatment of AD. However, the actual mechanism of cognitive-enhancing activity of WS-5 is still unclear and should be studied further.

## 5. Conclusions

Our study revealed that the WS-5 extract significantly attenuated the A*β*-induced memory impairment in mice that were evaluated by the passive avoidance test and the Morris water maze test. These results suggested that WS-5 is a promising therapeutic drug candidate for AD treatment and prevention. In addition, WS-5 showed substantial anti-AChE, antioxidative, anti-inflammatory, and anti-A*β* aggregation activities that could be further exploited as a valuable natural resource for the discovery and development of novel therapies in addition to the possible application in AD.

## Figures and Tables

**Table 1:** Quantification of compounds found in WS-5.

Sample	Compound	Equation	R^2^	Concentration (mg/g)
WS-5	Curcumin	y=31732x+41594	0.9905	7.123 ± 0.22
	6-Shogaol	y=3228.8x+14895	0.9998	1.896 ± 0.001
	Protocatechuic acid	y=4191.8x+4331.7	0.9999	0.432 ± 0.001

**Table 2:** Anti-AChE IC_50_ value of WS-5 and its marker compounds.

Extract or Compound	AChE activity
	^+^IC_50_ (*μ*g/ml)
WS-5	84.10
Curcumin	25.52
6-Shogaol	17.92
Protocatechuic acid	10.42

^+^The values indicate 50% AChE inhibitory effect and are the means of three independent experimental data sets.

## Data Availability

The data used to support the findings of this study are available from the corresponding author upon reasonable request.

## Abbreviations

AD: Alzheimer's disease
AChE: Acetylcholinesterase
A*β*: Amyloid-*β*
WS-5: 45 % ethanol extract of* Curcuma longa*,* Chaenomeles sinensis,* and* Zingiber officinale*
RG: Red ginseng
Cur: Curcumin
DPPH: 1, 1-diphenyl-2-picrylhydrazyl
TEM: Transmission electron microscopy
TAS: Total antioxidant status
DMEM: Dulbecco's Modified Eagle's Medium
CCK-8: Cell Counting Kit-8
DMSO: Dimethyl sulfoxide
ELISA: Enzyme-linked immunosorbent assay
PI: Protease inhibitor
FBS: Fetal bovine serum
BSA: Bovine serum albumin
TMB: 3, 3′, 5, 5′-Tetramethylbenzidine
LPS: Lipopolysaccharide
NO: Nitric oxide
IL-6: Interleukin-6
IL-1*β*: Interleukin-1 beta
TNF-*α*: Tumor necrosis factor alpha
ICV: Intracerebroventricular
HPLC: High-performance liquid chromatography
IC_50_: Concentration required for 50 % inhibition
DW: Distilled water
p: Level of significance
SEM: Standard error mean
ANOVA: Analysis of variance.

## Supplementary Materials

Supplementary Materials Figure S1: herbarium of WS-5 [*Curcuma longa* rhizome (A),* Chaenomeles sinensis* fruit (B), and* Zingiber officinale* rhizome (C)]. Figure S2: chemical structure of 6-gingerol. Figure S3: HPLC chromatogram showing the quantification of WS-5 with 6-gingerol compound. Chromatogram: (A) standard compound and (B) WS-5 extract. Table S1: quantification of 6-gingerol found in WS-5.

## References

[1] Evin G., Weidemann A. (2002). Biogenesis and metabolism of Alzheimer's disease A*β* amyloid peptides. *Peptides*.

[2] Thies W., Bleiler L. (2013). Alzheimer's disease facts and figures. *Alzheimer's & Dementia*.

[3] Hardy J., Selkoe D. J. (2002). The amyloid hypothesis of Alzheimer's disease: progress and problems on the road to therapeutics. *Science*.

[4] Zhang C. (2012). Natural compounds that modulate BACE1-processing of amyloid-beta precursor protein in Alzheimer's disease. *Discovery Medicine*.

[5] Gupta S. k., Sharma A. (2014). Medicinal properties of *Zingiber officinale* Roscoe - A Review. *IOSR Journal of Pharmacy and Biological Sciences*.

[6] Anosike C. A., Obidoa O., Ezeanyika L. U. S., Nwuba M. M. (2009). Anti-inflammatory and anti-ulcerogenic activity of the ethanol extract of ginger (*Zingiber officinale*). *African Journal of Biochemistry Research*.

[7] Mathew M., Subramanian S. (2014). In vitro evaluation of anti-Alzheimer effects of dry ginger (*Zingiber officinale* Roscoe) extract. *Indian Journal of Experimental Biology (IJEB)*.

[8] Choi J. G., Kim S. Y., Jeong M., Oh M. S. (2018). Pharmacotherapeutic potential of ginger and its compounds in age-related neurological disorders. *Pharmacology & Therapeutics*.

[9] Kim J. E., Jo Y. J., Leem J. Y. (2015). The acetylcholinesterase inhibitory activity of the EtOH extract of chaenomelis fructus and its effects on the metabolism of amyloid precursor protein in neuroblastoma cells. *Korean Journal of Pharmacognosy*.

[10] Kim J. H., An C. W., Kim Y. J. (2018). Antioxidant activity of n-butanol fraction of *Chaenomeles sinensis* fruit in caenorhabditis elegans. *Korean Journal of Pharmacognosy*.

[11] Sancheti S., Seo S.-Y. (2013). Antidiabetic and antiacetylcholinesterase effects of ethyl acetate fraction of *Chaenomeles sinensis* (Thouin) Koehne fruits in streptozotocin-induced diabetic rats. *Experimental and Toxicologic Pathology*.

[12] Han Y., Kim Y., Natarajan S. (2016). Antioxidant and anti-inflammatory effects of *Chaenomeles sinensis* leaf extracts on lps-stimulated raw 264.7 cells. *Molecules*.

[13] Sawai-Kuroda R., Kikuchi S., Shimizu Y. K. (2013). A polyphenol-rich extract from *Chaenomeles sinensis* (Chinese quince) inhibits influenza A virus infection by preventing primary transcription in vitro. *Journal of Ethnopharmacology*.

[14] Ahmed T., Gilani A.-H. (2013). Therapeutic potential of turmeric in Alzheimer's disease: Curcumin or curcuminoids?. *Phytotherapy Research*.

[15] Lee W.-H., Loo C.-Y., Bebawy M., Luk F., Mason R. S., Rohanizadeh R. (2013). Curcumin and its derivatives: their application in neuropharmacology and neuroscience in the 21st century. *Current Neuropharmacology*.

[16] Mehrotra S., Agnihotri G., Singh S., Jamal F. (2013). Immunomodulatory potential of *Curcuma longa*: a review. *South Asian Journal of Experimental Biology*.

[17] Labban L. (2014). Medicinal and pharmacological properties of turmeric (*Curcuma longa*): a review. *International Journal of Pharmacy and Biomedical Sciences*.

[18] Zhang L., Fang Y., Xu Y. (2015). Curcumin improves amyloid *β*-peptide (1-42) induced spatial memory deficits through BDNF-ERK signaling pathway. *PLoS ONE*.

[19] Mishra S., Palanivelu K. (2008). The effect of curcumin (turmeric) on Alzheimer's disease: an overview. *Annals of Indian Academy of Neurology*.

[20] Thangthaeng N., Miller M., Poulose S. M., Bielinski D., Fisher D., Hale B. S. (2015). Differential effects of blueberry polyphenols on age-associated neuroinflammation and cognition. *The FASEB Journal*.

[21] Leem J. Y., Kim J. E., Kim H. S., Shrestha A. C., Ham H. N. Korean Patent Pending.

[22] Lee H. Y., Weon J. B., Jung Y. S., Kim N. Y., Kim M. K., Ma C. J. (2016). Cognitive-enhancing effect of *Aronia melanocarpa* extract against memory impairment induced by scopolamine in mice. *Evidence-Based Complementary and Alternative Medicine*.

[23] Murray A. P., Faraoni M. B., Castro M. J., Alza N. P., Cavallaro V. (2013). Natural AChE inhibitors from plants and their contribution to Alzheimer’s disease therapy. *Current Neuropharmacology*.

[24] Ramassamy C. (2006). Emerging role of polyphenolic compounds in the treatment of neurodegenerative diseases: a review of their intracellular targets. *European Journal of Pharmacology*.

[25] Terry A. V., Buccafusco J. J. (2003). The cholinergic hypothesis of age and Alzheimer's disease-related cognitive deficits: recent challenges and their implications for novel drug development. *The Journal of Pharmacology and Experimental Therapeutics*.

[26] Masters C. L., Selkoe D. J. (2012). Biochemistry of amyloid *β*-protein and amyloid deposits in Alzheimer disease. *Cold Spring Harbor Perspective in Medicine*.

[27] Yanagisawa D., Taguchi H., Morikawa S. (2015). Novel curcumin derivatives as potent inhibitors of amyloid *β* aggregation. *Biochemistry and Biophysics Reports*.

[28] Kim D. W., Lee S. M., Woo H. S. (2016). Chemical constituents and anti-inflammatory activity of the aerial parts of *Curcuma longa*. *Journal of Functional Foods*.

[29] Sutalangka C., Wattanathorn J. (2017). Neuroprotective and cognitive-enhancing effects of the combined extract of *Cyperus rotundus* and *Zingiber officinale*. *BMC Complementary and Alternative Medicine*.

[30] Mahdy K. A., Gouda N. A. M., Marrie A. H. (2014). Protective effect of ginger (*Zingiber officinale*) on Alzheimers disease induced in rats. *Journal of Neuroinfectious Diseases*.

[31] Wang J., Hu S., Nie S., Yu Q., Xie M. (2016). Reviews on mechanisms of *in vitro* antioxidant activity of polysaccharides. *Oxidative Medicine and Cellular Longevity*.

[32] Manoharan S., Guillemin G. J., Abiramasundari R. S., Essa M. M., Akbar M., Akbar M. D. (2016). The role of reactive oxygen species in the pathogenesis of alzheimer's disease, parkinson's disease, and huntington's disease: a mini review. *Oxidative Medicine and Cellular Longevity*.

[33] Liu Y. H., Giunta B., Zhou H.-D., Tan J., Wang Y. J. (2012). Immunotherapy for Alzheimer disease-the challenge of adverse effects. *Nature Reviews Neurology*.

[34] Yang F., Lim G. P., Begum A. N. (2005). Curcumin inhibits formation of amyloid *β* oligomers and fibrils, binds plaques, and reduces amyloid in vivo. *The Journal of Biological Chemistry*.

[35] Ahmad E., Ahmad A., Singh S., Arshad M., Khan A. H., Khan R. H. (2011). A mechanistic approach for islet amyloid polypeptide aggregation to develop anti-amyloidogenic agents for type-2 diabetes. *Biochimie*.

[36] Hasanbašić S., Jahić A., Berbić S., Žnidarič M. T., Žerovnik E. (2018). Inhibition of protein aggregation by several antioxidants. *Oxidative Medicine and Cellular Longevity*.

[37] Lull M. E., Block M. L. (2010). Microglial activation and chronic neurodegeneration. *Neurotherapeutics*.

[38] Kuno R., Wang J., Kawanokuchi J., Takeuchi H., Mizuno T., Suzumura A. (2005). Autocrine activation of microglia by tumor necrosis factor-*α*. *Journal of Neuroimmunology*.

[39] Tansey M. G., McCoy M. K., Frank-Cannon T. C. (2007). Neuroinflammatory mechanisms in Parkinson's disease: potential environmental triggers, pathways, and targets for early therapeutic intervention. *Experimental Neurology*.

[40] Eun C.-S., Lim J.-S., Lee J., Lee S.-P., Yang S.-A. (2017). The protective effect of fermented *Curcuma longa* L. on memory dysfunction in oxidative stress-induced C6 gliomal cells, proinflammatory-activated BV2 microglial cells, and scopolamine-induced amnesia model in mice. *BMC Complementary and Alternative Medicine*.

[41] Kwon Y. K., Choi S. J., Kim C. R. (2015). Effect of *chaenomeles sinensis* extract on choline acetyltransferase activity and trimethyltin-induced learning and memory impairment in mice. *Chemical & Pharmaceutical Bulletin*.

[42] Prasansuklab A., Tencomnao T. (2013). Amyloidosis in alzheimer’s disease: the toxicity of amyloid beta (A*β*), mechanisms of its accumulation and implications of medicinal plants for therapy. *Evidence-Based Complementary and Alternative Medicine*.

[43] Foerde K., Shohamy D. (2011). The role of the basal ganglia in learning and memory: insight from Parkinson's disease. *Neurobiology of Learning and Memory*.

[44] Murali Doraiswamy P. (2002). Non-cholinergic strategies for treating and preventing Alzheimer's disease. *CNS Drugs*.

[45] Joseph J. A., Denisova N. A., Arendash G. (2003). Blueberry supplementation enhances signaling and prevents behavioral deficits in an Alzheimer disease model. *Nutritional Neuroscience*.

[46] Bachurin S. (2003). Medicinal chemistry approaches for the treatment and prevention of Alzheimer's disease. *Medicinal Research Reviews*.

