# Corrigendum to “WS-5 Extract of *Curcuma longa, Chaenomeles sinensis*, and *Zingiber officinale* Contains Anti-AChE Compounds and Improves *β*-Amyloid-Induced Memory Impairment in Mice”

**DOI:** 10.1155/2021/5127585

**Published:** 2021-08-10

**Authors:** Ju Eun Kim, Abinash Chandra Shrestha, Hyo Shin Kim, Ha Neul Ham, Jun Hyeong Kim, Yeong Jee Kim, Yun Jeong Noh, Su Jin Kim, Dae Keun Kim, Hyung Kwon Jo, Dae Sung Kim, Moon Kwang Hyun, Jeong Ho Lee, Kyung Ok Jeong, Jae Yoon Leem

**Affiliations:** ^1^College of Pharmacy, Woosuk University, Wanju, Jeonbuk 55338, Republic of Korea; ^2^Hanpoong Pharm. Co., Ltd., Wanju, Jeonbuk 55336, Republic of Korea; ^3^Sunchang Institute of Health and Longevity, Sunchang, Jeonbuk 56015, Republic of Korea

In the article titled “WS-5 Extract of *Curcuma longa, Chaenomeles sinensis,* and *Zingiber officinale* Contains Anti-AChE Compounds and Improves *β*-Amyloid-Induced Memory Impairment in Mice” [1], there was an error in Figure 1(a), where “DPPH inhibition activity (% of control)” should be corrected to “DPPH radical level (% of control).”

In addition, the authors wish to replace Figure 2 to show a modified possible underlying mechanism for WS-5.

The corrected Figures 1 and 2 are shown as follows.

## Figures and Tables

**Figure 1:**
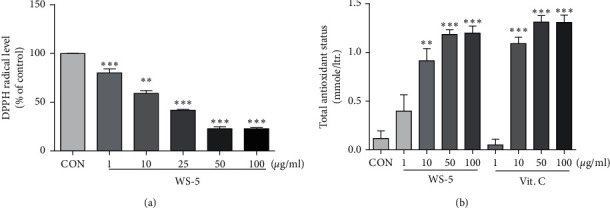
DPPH radical scavenging activity and TAS of WS-5. Inhibition of DPPH was measured using the radical scavenging assay (a), with vitamin C as positive control, along with the TAS assay (b). Results are expressed as the means ± SEM of three independent experiments (^*∗*^*p* < 0.05, ^*∗∗*^*p* < 0.01, ^*∗∗∗*^*p* < 0.001*vs*. control).

**Figure 2:**
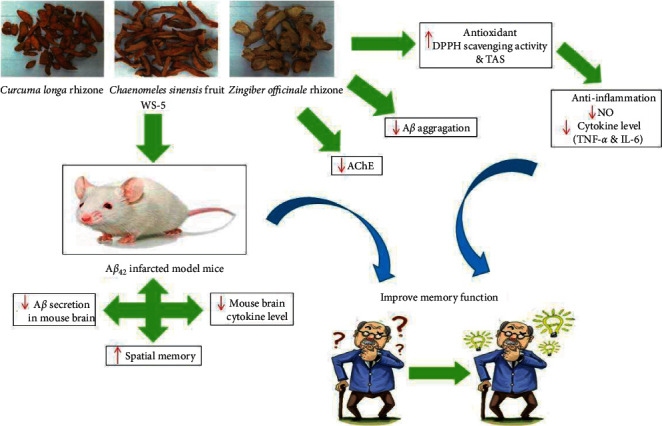
Schematic diagram showing possible underlying mechanism of WS-5.
