# Supplementary material for: WS-5 Extract of Curcuma longa, Chaenomeles sinensis, and Zingiber officinale Contains Anti-AChE Compounds and Improves β-Amyloid-Induced Memory Impairment in Mice
Source: Evid Based Complement Alternat Med. 2019 Apr 1;2019:5160293. doi: 10.1155/2019/5160293 (PMC6463685; doi:10.1155/2019/5160293)
Supplement: Supplementary Materials — Figure S1: herbarium of WS-5 [Curcuma longa rhizome (A), Chaenomeles sinensis fruit (B), and Zingiber officinale rhizome (C)]. Figure S2: chemical structure of 6-gingerol. Figure S3: HPLC chromatogram showing the quantification of WS-5 with 6-gingerol compound. Chromatogram: (A) standard compound and (B) WS-5 extract. Table S1: quantification of 6-gingerol found in WS-5. [file 5160293.f1.docx]

**WS-5 extract of *Curcuma longa***, ***Chaenomeles sinensis* and** ***Zingiber officinale* contains anti-AChE compounds and improves β-amyloid-induced memory impairment in mice**

**Ju Eun Kim et al.**

**Supplementary Material**

**Material and Methods**

**Collection and identification of Plant materials**

The fresh rhizomes of *C. longa* L, *Z. officinale* Roscoe, and fruits of *C. sinensis* Koehne and Red ginseng were purchased from HeungIl Pharmaceutical Co. (Seoul, Korea). The plant specimen were identified compared with the literatures and authenticated by Professor Dae Keun Kim, Department of Pharmacy, Woosuk University. A voucher specimen (WSU-18-002) has been deposited in the herbarium of the College of Pharmacy, Woosuk University, South Korea (Figure S1).

**Quantification of WS-5 by HPLC analysis**

The quantitative analysis of 6-gingerol from WS-5 was detected using the HPLC system Shiseido with photodiode array detector. The elution profile was monitored at 254 nm using a column temperature of 40 ºC. Chromatography was performed using a Capcell Pak C18 MG II (250 Χ 4.6 mm, 5 μM; Shiseido Co., Ltd., Tokyo, Japan). The mobile phase was composed of 0.1% trifluoroacetic acid in water (solvent A) and 0.1% trifluoroacetic acid in acetonitrile (solvent B). The gradient program was 0-10 min, 100% of solvent A; 10-13 min, 90% of solvent A; 13-20 min, 60% of solvent A; 20-25 min, 60% of solvent A; 25-35 min, 50% of solvent A; 35-40 min, 50% of solvent A; 40-45 min, 55% of solvent A; 45-55 min, 30% of solvent A; 55-60 min, 30% of solvent A with a flow rate of 1 ml/min and the injection volume was 10 μl. Chemical structure of 6-gingerol, HPLC chromatogram of WS-5 with 6-gingerol and quantification table of 6-gingerol are shown in supplementary Figure S2, S3 and Table S1.

**
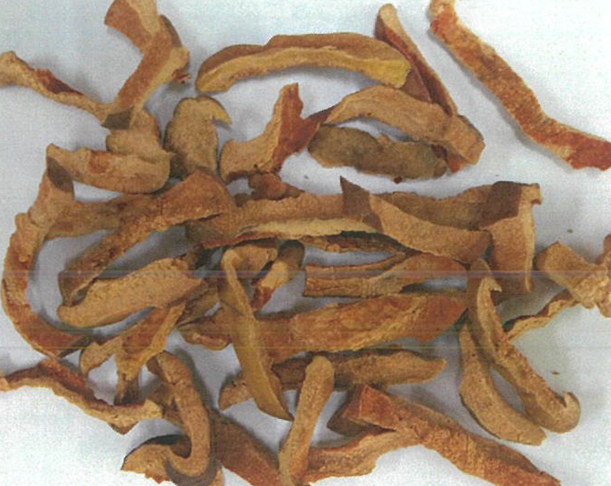

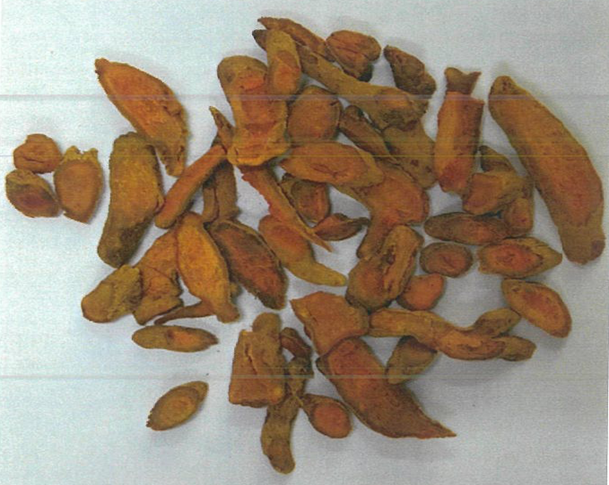
**

B

A

*Curcuma longa* rhizome *Chaenomeles sinensis* fruit


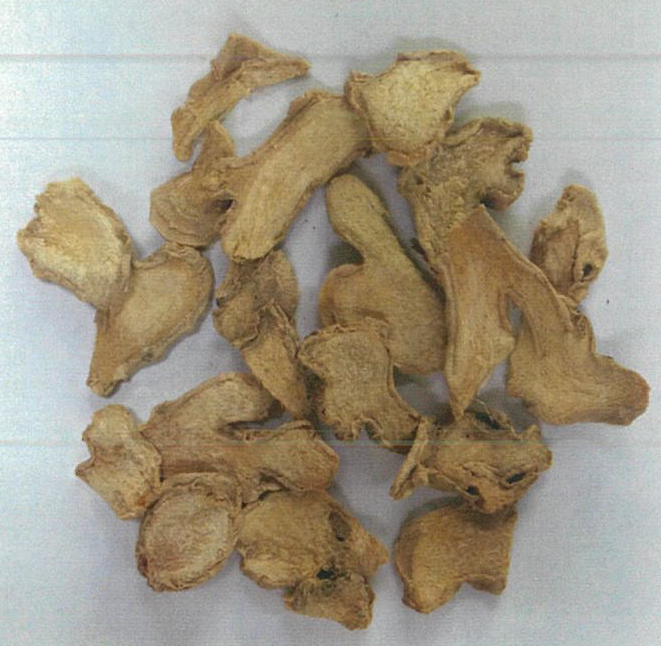


C

*Zingiber officinale* rhizome

| **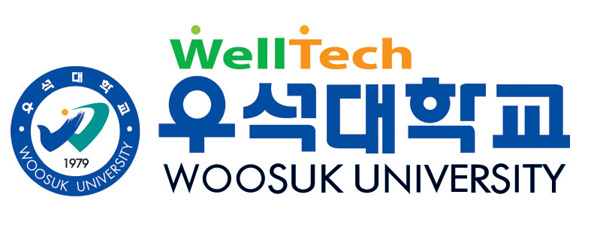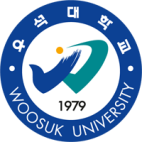Scientific Name**: *Curcuma longa*, *Chaenomeles sinensis* and *Zingiber officinale.*  **Parts used**: rhizome, fruit and rhizome.  **Specimen No.**: WSU-18-002  **Identified by**: Prof. Dae Keun Kim |
| --- |

**Figure S1:** **Herbarium of WS-5 [*Curcuma longa* rhizome (A), *Chaenomeles sinensis* fruit (B) and *Zingiber officinale* rhizome (C)].**

A

**Figure S2. Chemical structure of 6-gingerol.**

**A**


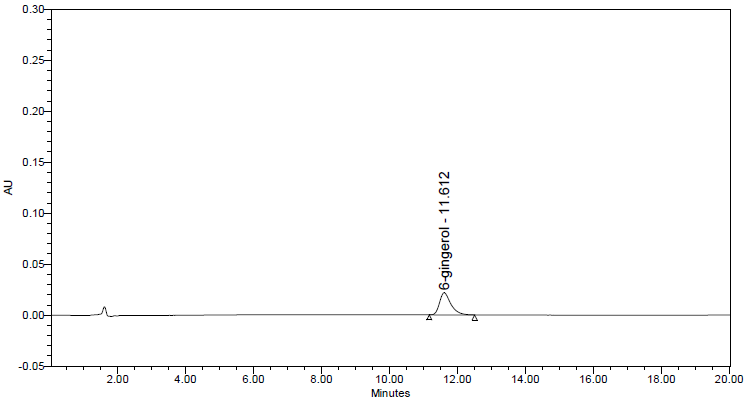


**B**


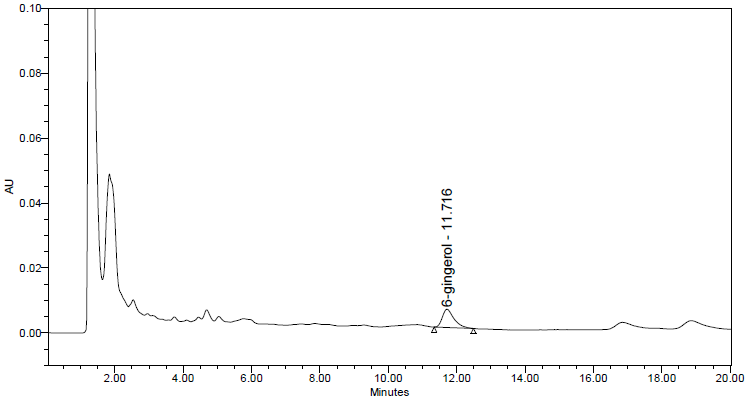


**Figure S3: HPLC chromatogram showing the quantification of WS-5 with 6-gingerol compound. Chromatogram: A) Standard compound and B) WS-5 extract.**

**Table S1. Quantification of 6-gingerol found in WS-5**

| Sample | Compound | Regression equation^a^ | Linearity (R^2^) | Concentration (mg/g) |
| --- | --- | --- | --- | --- |
| WS-5 | 6-gingerol | *y* = 3554.3*x* + 23769 | 0.9997 | 4.704 |

^a^ Where Y is a peak area and X is the concentration of the analyzed material.
